# Delivery room resuscitation of conjoined twins

**DOI:** 10.1038/s41372-026-02735-5

**Published:** 2026-05-27

**Authors:** K. Taylor Wild, Natalie E. Rintoul, Anne M. Ades, Nahla Khalek, Juliana S. Gebb, Edward R. Oliver, Tom Reynolds, Leny Mathew, Heidi M. Herrick, Kelle Matthews, Lauren Heimall, Olivia Nelson, Benjamin B. Bruins, Jack Rychik, Emily A. Partridge, Alan W. Flake, Holly L. Hedrick

**Affiliations:** 1 Division of Neonatology, Children’s Hospital of Philadelphia, Perelman School of Medicine at University of Pennsylvania, Philadelphia, PA USA; 2 Richard D. Wood Center for Fetal Diagnosis and Treatment, Children’s Hospital of Philadelphia, Philadelphia, PA USA; 3 Division of Pediatric General, Thoracic, and Fetal Surgery, Children’s Hospital of Philadelphia, Perelman School of Medicine at University of Pennsylvania, Philadelphia, PA USA; 4 Department of Radiology, Children’s Hospital of Philadelphia, Perelman School of Medicine at University of Pennsylvania, Philadelphia, PA USA; 5 Department of Anesthesiology and Critical Care Medicine, Children’s Hospital of Philadelphia, Perelman School of Medicine at University of Pennsylvania, Philadelphia, PA USA; 6 Division of Cardiology, Children’s Hospital of Philadelphia, Perelman School of Medicine at University of Pennsylvania, Philadelphia, PA USA

**Keywords:** Paediatrics, Palliative care

## Abstract

**Objective:**

Describe the golden hour and hospital outcomes of conjoined twins

**Methods:**

Retrospective single center study of conjoined twins. Main outcome measures included delivery room characteristics. Secondary outcomes included survival and length of stay.

**Results:**

From 2013-2025, there were 19 sets of conjoined twins (active resuscitation in 13; palliative in 6). Respiratory interventions in the delivery room were frequently needed including continuous positive airway pressure (75%), positive pressure ventilation (67%), and endotracheal intubation (25%). There were two sets with emergency separation, each with one surviving twin. For the 10 sets who underwent separation, survival at NICU discharge was 80% (twin A) and 100% (twin B).

**Conclusion:**

The delivery room resuscitation of conjoined twins is complex with high rates of advanced respiratory intervention. These deliveries necessitate an experienced multidisciplinary team and an individualized delivery plan based on specific anatomy and reinforced with simulation. Survival is high among twins who undergo separation.

## Introduction

Conjoined twins are rare and occur in 1 in every 50–60,000 pregnancies. When accounting for fetal deaths, terminations, and stillbirths, the final incidence is ~1 in 250,000 live births with a female predominance of 3:1 [1]. Conjoined twins are generally classified into eight major types based on the most prominent site of fusion, including: cephalopagus, thoracopagus, omphalopagus, ischiopagus, parapagus, craniopagus, pyopagus, and rachipagus [2].

Conjoined twins are typically delivered via cesarean sometime between 32 and 35 weeks gestational age, primarily due to size constraints, risk of stillbirth, and to minimize maternal surgical risk. The biggest challenges for the delivery room resuscitation of conjoined twins include the coordination of bedside clinical teams for each infant, environmental considerations (significant spatial constraints), and the resuscitation needs of the infants (providing respiratory support, obtaining vascular access, and maintaining thermoregulation). While the Neonatal Resuscitation Program (NRP) from the American Academy of Pediatrics provides a standardized and evidence-based approach to delivery room resuscitation [3], there are no standard guidelines for the delivery room resuscitation of conjoined twins. Yamada et al. proposed a modified algorithm of NRP based on the experience of preparing for and completing the delivery room resuscitation of a set of omphaloischiopagus twins [1, 4], but these deliveries are each unique and teams must be prepared for a wide range of clinical scenarios and potential complications/challenges. This study reviewed the delivery room experience at our center and describes the golden hour care, immediate delivery room resuscitative interventions/care needs, and short-term outcomes to first discharge in this unique population.

## Methods

### Design

We conducted a retrospective single center cohort study of all conjoined twins born in the Garbose Family Special Delivery Unit at the Children’s Hospital of Philadelphia from 2013 to 2025. Study data were sourced from the Clinical Outcomes Data Archive (CODA) Registry [5]. The CHOP Institutional Review Board approved this study (IRB 21-018553) with a waiver of informed parental consent.

### Hospital delivery room resuscitation protocols

Delivery room interventions to stabilize the airway, obtain intravenous access (peripheral and/or umbilical), maintain thermoregulation, and perform any other procedures take place in an infant resuscitation room immediately adjacent to the operative delivery room. The neonatal Special Delivery Unit resuscitation team typically includes a neonatologist, a neonatal fellow physician, an advanced practice provider, two to three neonatal intensive care unit (NICU) nurses, and a respiratory therapist. For deliveries of conjoined twins, there is a second team consisting of a second neonatologist, advanced practice provider, respiratory therapist, and an additional two to three nurses. The goal of a very specific set-up is to achieve appropriate coordination of bedside clinicians for each infant in the setting of significant spatial constraints and the necessary coordination to provide respiratory support, obtain vascular access, and maintain thermoregulation. Each twin has an individual team and each twin has different colored Coban™ self-adherent wrap around all medical devices (pulse oximetry probes, electrocardiogram leads, intravenous pumps, stethoscopes, airway equipment, etc).

### Hospital delivery planning

A multidisciplinary team reviews deliveries in a biweekly delivery room planning meeting and discusses special considerations for each set of twins. We develop a 3-D model and run simulations to adjust our standard conjoined twin guidelines if needed based on the positioning and anatomy of the twins and with the teams expected to be at the delivery. Monthly video review conferences review prior deliveries for quality improvement and education and have been used to continue to improve our delivery room set-up.

### Main outcome measures

Main outcome measures included delivery room characteristics, in particular respiratory interventions. Secondary outcomes included survival and length of stay.

### Data analysis

Due to the descriptive nature of this study and expected small sample sizes, we report a description of the summary statistics without any formal statistical analysis. We conducted all analyses in R V.4.1.2.

## Results

From 2013 to 2025, there were 19 sets of conjoined twins born in the Garbose Family Special Delivery Unit at the Children’s Hospital of Philadelphia. Table 1 summarizes prenatal characteristics. Thoracopagus (47.4%, *N* = 9) and omphalopagus (21.1%, *N* = 4) were the most common types of conjoined twins. Based on prenatal imaging, separation was predicted to be possible in nine sets, potentially possible in three sets, and not possible in seven sets. Deliveries occurred urgently on nights/weekends in >30% of cases. Table 2 summarizes neonatal and delivery room outcomes. There were 13 sets of twins who had an active resuscitation, including two sets of twins who had active resuscitations despite a prenatal determination that separation would not be possible, and six sets of twins who had a planned non-intervention palliative delivery. Two sets of twins had emergency separation surgery immediately following birth (one secondary to instability, one planned prenatally). One case involved omphalopagus twins in which there was associated concern for OEIS spectrum in twin A with giant omphalocele, severe pulmonary hypoplasia, kyphoscoliosis, and bladder exstrophy. The twins were delivered at 29 weeks’ gestation in the setting of preterm rupture of membranes and non-reassuring fetal status of twin A. Both twins were intubated in the delivery room, but given significant hemodynamic instability and concern for imminent demise in twin A, emergency separation was performed. Following surgery, twin A continued with severe hemodynamic instability and the family redirected goals of care at about 18 h of life. Twin B had a successful ileostomy for an imperforate anus and was discharged home after 110 days. In the second emergency separation, thoracopagus twins were born at 34 weeks’ gestation. Twin B was known prenatally to have severe cardiac anomalies with absence of ventricular outflow tracts such that he was completely supported by arterial circulation coming from twin A’s aorta. Given the determination that twin B’s anomalies were incompatible with life, even if they remained conjoined, there were prenatal discussions about immediate separation with reconstruction of twin A. The twins were immediately separated following birth with demise of twin B and successful discharge of twin A at ~1 month of life.

**Table 1:** Prenatal characteristics (*N* = 19).

	Median (IQR), *N*(%)
Maternal age at birth (years)	32.3 (28.5, 34.6)
Gravidity	3 (2, 5)
Parity	2 (1, 3)
Mode of delivery (cesarean)	19 (100%)
Relationship status	
Married and living with partner	16 (84.2%)
Unmarried and not living with partner	3 (15.8%)
Level of education	
Completed college	8 (42.1%)
Completed high school	4 (21.1%)
Master’s degree or greater	3 (15.8%)
Some college	4 (21.1%)
Self-reported race/ethnicity	
Non Hispanic White	11 (57.9%)
Non Hispanic Black	4 (21.1%)
Non Hispanic Other	1 (5.3%)
Hispanic	1 (5.3%)
Missing	2 (10.5%)
Insurance status	
Commercial	9 (47.4%)
Government	6 (31.6%)
Self-pay	4 (21.1%)
Gestational age at time of initial imaging (weeks)	23.4 (20.5, 28.0)
Type of conjoined twin	
Cephalopagus	1 (5.3%)
Craniopagus	1 (5.3%)
Ischiopagus	1 (5.3%)
Omphalopagus	4 (21.1%)
Parapagus	1 (5.3%)
Pygopagus	1 (5.3%)
Ischiopagus	1 (5.3%)
Thoracopagus	9 (47.4%)
Received betamethasone	13 (68.4%)
Predicted separation	
Yes	9 (47.4%)
Potentially	3 (15.8%)
No	7 (36.8%)
Night/Weekend Delivery (5 pm-8 am or weekends)	6 (31.6%)

*IQR* interquartile range, *SD* standard deviation.

**Table 2:** Neonatal Characteristics.

	Median (IQR), *N*(%)	Median (IQR), *N*(%)
	Twin A (*N* = 19)	Twin B (*N* = 19)
Gestational Age at birth (weeks)	32.3 (31.4, 34.4)	32.3 (31.4, 34.4)
Sex		
Female	13 (68.4%)	13 (68.4%)
Male	6 (31.6%)	6 (31.6%)
Birthweight (kg)	2.0 (1.5, 2.3)	2.0 (1.4, 2.4)
Planned non-intervention palliative delivery	6 (31.6%)	6 (31.6%)

Delivery Room Outcomes for Infants with Active Delivery Room Resuscitation^a^		
	**Twin A (*N* = 12)**	**Twin B (*N* = 12)**
1 min Apgar	7 (3, 8)	7 (4, 8)
5 min Apgar	7 (5, 9)	8 (5, 8)
Time to Pre-ductal SpO_2_ > 85% (minutes)	7 (5, 37)	8 (5, 63)
HR > 100 during resuscitation		
Yes	12 (100%)	12 (100%)
Time to HR > 100 beats per minute (minutes)	5 (0, 14)	2 (0, 6)
Respiratory Interventions		
Continuous positive airway pressure	9 (75.0%)	9 (75.0%)
Positive pressure ventilation	8 (66.7%)	7 (58.3%)
Intubation/mechanical ventilation	3 (25.0%)	3 (25.0%)
Laryngeal mask	1 (8.3%)	1 (8.3%)
Final Mode of Respiratory Support		
No supplemental respiratory support	6 (50.0%)	3 (25.0%)
Continuous positive airway pressure	5 (41.7%)	7 (58.3%)
Intubation/mechanical ventilation	1 (8.3%)	2 (16.7%)
Last FiO_2_ in Delivery Room	30 (21, 100)	30 (21, 100)
Normal Saline Bolus Given in Delivery Room	2 (16.7%)	2 (16.7%)
First temperature (degrees Celsius)	36.2 (34.7, 37.3)	36.2 (35.2, 36.8)
Last temperature (degrees Celsius)	36.4 (36.1, 36.7)	36.5 (35.9, 36.7)
Minutes to vascular access	24.0 (17.8, 26.3)	28.0 (24.0, 31.3)
Peripheral intravenous access	10 (83.3%)	9 (75.0%)
Umbilical venous catheter	3 (25.0%)	3 (25.0%)
Umbilical arterial catheter	1 (8.3%)	1 (8.3%)

*IQR* interquartile range, *SD* standard deviation.

^a^Delivery room data missing for one set of twins who went immediately for separation surgery following birth.

**Fig. 1:**
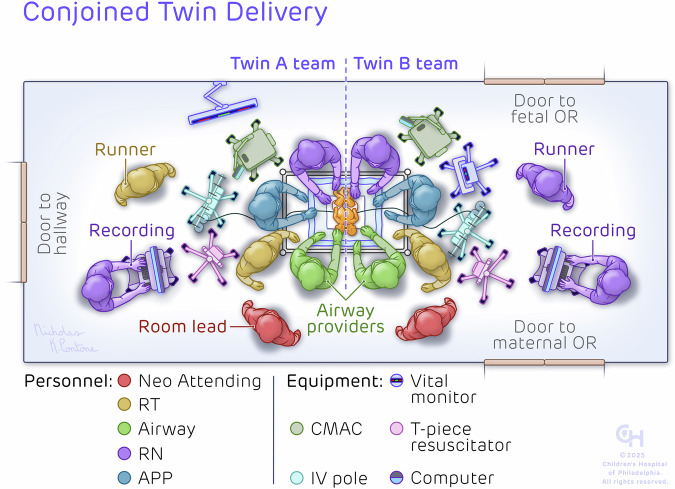
Delivery room set-up to achieve appropriate coordination of bedside clinicians for each infant.

Prematurity was common with a median gestational age of 32.3 weeks (interquartile range (IQR) 31.4, 34.4 weeks). There was no difference in gestational age between infants who had an active resuscitation versus a non-intervention palliative delivery. Respiratory interventions were frequently needed in the delivery room and included continuous positive airway pressure (75.0%), positive pressure ventilation (66.7%), and endotracheal intubation (25.0%). Laryngeal masks were used in 8.3% for continuous positive airway pressure and/or positive pressure ventilation. There were no deliveries that needed an otolaryngologist or advanced airway team. While there were similar rates of interventions needed throughout the resuscitation, there was one case of differential need for intubation and mechanical ventilation between twins in the case of an emergency separation. Figure 1 shows our delivery room set-up that optimizes team and equipment positioning for best access to each twin. Vascular access varied with umbilical line placement in 25%. First temperature was 36.2 degrees Celsius with a final temperature of 36.4 degrees Celsius in twin A and 36.5 degrees Celsius in twin B. Neonatal outcomes are shown in Table 3. As noted above, there were two sets of twins where emergency separation was performed, each with one surviving twin. Separation was performed in 10/13 (76.9%) sets of conjoined twins who had an active resuscitation at a median of 251 days (IQR 25, 324). Separation was not possible in 3/13 (23.0%) sets, including two sets where separation had been deemed not possible prenatally, with no survivors. Among the 10 sets who underwent separation, survival rate at NICU discharge was 80% for twin A and 100% for twin B. Length of stay among survivors was >300 days.

**Table 3:** Neonatal Outcomes.

	Median (IQR), *N*(%)	Median (IQR), *N*(%)
	Twin A (*N* = 19)	Twin B (*N* = 19)
Planned non-intervention palliative delivery	6 (31.6%)	6 (31.6%)

Outcomes of infants with an active resuscitation		
	**Twin A (*N* = 13)**	**Twin B (*N* = 13)**
Length of stay among survivors (days)	329 (260, 375)	305 (100, 369)
Circumstances of separation among infants with an active resuscitation		
Non-emergent/delayed separation	8 (61.5%)	8 (61.5%)
Emergency/immediate separation	2 (15.3%)	2 (15.3%)
Non-separable^a^	3 (23.0%)	3 (23.0%)
Survival at NICU discharge among all infants with active resuscitation (*N* = 13)	8 (66.7%)	10 (83.3%)
Survival at NICU discharge among infants who underwent separation (*N* = 10)	8 (80.0%)	10 (100.0%)
Age at separation (days)	251 (25, 324)	251 (25, 324)

*IQR* interquartile range, *SD* standard deviation.

^a^2 sets deemed non-separable prenatally.

## Discussion

The delivery room resuscitation of conjoined twins is complex and requires considerable coordination of experienced and cohesive multidisciplinary teams. Because these deliveries are exceedingly rare, this cohort aims to address the limited data on delivery room resuscitation. In our cohort, conjoined twins were likely to be born prematurely at a median of 32.3 weeks. As a result of preterm delivery and the unique anatomical challenges of each individual set of conjoined twins, there were relatively high rates of respiratory intervention in the delivery room including continuous positive airway pressure (75.0%), positive pressure ventilation (66.7%), endotracheal intubation (25.0%), and laryngeal mask placement (8.3%), with a trend toward increased need for respiratory support with higher level connections.

There are no standard guidelines for these highly unique deliveries which necessitate important deviations from NRP. Teams must anticipate many clinical scenarios and potential complications, including the potential need for emergency separation, which occurred in two sets in this cohort. Preparation, particularly with multidisciplinary simulation, is essential. Clinicians must develop an individualized delivery plan based on the specific anatomy of each set of twins and should include simulation of the resuscitation prior to delivery, ^1,4,6,7^ especially since >30% of deliveries occurred on nights and/or weekends. This includes delivery room set-up to accommodate spatial constraints, discussions about how interventions will be performed and documented, especially respiratory interventions and intravenous access, and how to calculate the weight and administer medications. For example, if there are vascular connections, medications administered to one twin will be distributed to both, as will be discussed further below. Although they are one set of twins at the time of delivery, each twin is treated as an individual. To allow for rapid and accurate identification of each twin, we color code equipment and personnel with Coban™ self-adherent wrap with one color for each twin [6, 7].

Administering non-invasive respiratory support and positioning for endotracheal intubation can be challenging, particularly in infants with thoracic connections. A portable video laryngoscope can sometimes be helpful to overcome space issues to facilitate endotracheal intubation. In our cohort, all intubated infants had thoracic connections. When the faces are close together and positive pressure ventilation is needed, teams may find laryngeal masks useful in lieu of facial masks as they are less bulky and are not dependent on achieving a facial seal. It may be helpful, and sometimes even necessary, to hold one twin above the other to allow for alignment of the head and neck of the other twin if endotracheal intubation is needed. However, with this maneuver, there is the risk that one twin may develop relative anemia and the other relative polycythemia in the setting of a shared vascular system and/or that the inferior vena cava might get compressed by the increased liver mass if the prone twin is not suspended properly. While about half of infants in our cohort received either non-invasive or invasive ventilation of some type, our rates of respiratory intervention were lower than those previously described. Snoap et al. reported their experience with five sets of conjoined twins in which all required non-invasive or invasive ventilation, including endotracheal intubation in 6/10 infants in the setting of having an otolaryngologist present at all conjoined twin deliveries [8]. In contrast, our center consults otolaryngology only in the case of airway risk factors, and otolaryngologists were not present in the deliveries described here. This difference in prevalence of respiratory interventions may reflect practice differences between specialties. Neonatologists have extensive experience supporting infants with non-invasive support during complicated delivery room perinatal transitions and may be more likely to defer intubation. In the case described by Yamada et al., the twins delivered at 32 6/7 weeks’ gestation, and both received continuous positive airway pressure and positive pressure ventilation, but no medications or intravascular access in the delivery room [1].

Obtaining vascular access can be difficult and requires careful planning in terms of team member and equipment placement. The umbilical cords may be shared and/or inaccessible, so infants often need peripheral venous and arterial access. Teams must be prepared for these and other challenging scenarios. For instance, some twins have limited shared organs but have large vasculature connections that can complicate the administration of medications. For example, if one twin requires intubation and receives pre-intubation medications with sedation and neuromuscular blockade, the team must be prepared to support the other twin who will also be affected by these medications in the setting of shared vasculature. Careful review of prenatal imaging by the prenatal and postnatal teams ensures physiologic optimization during the perinatal transition and beyond. In our cohort and others, we had relatively equivalent rates of respiratory interventions between twins [1, 8], supporting that if intubation was performed in one twin, it was also typically performed in the other. Other specific considerations include determining the potential degree of shared diaphragm and sternum as well as individual and combined respiratory mechanics between twins and how they may impact each other. It is also important to note if the heart rates are the same on electrocardiogram tracings and to distinguish separate heart rates between twins, if possible. If the infants are relatively similar appearing in weight, then medication dosing is generally administered using weight-based dosing by taking the combined weight of both twins and dividing it in half. Medications are then administered to each twin based on an individual dosing weight. However, it is important to note if there are significant growth discrepancies and adjust accordingly. Individual sets of twins will have different vascular connections and potential drug crossover, and the team may need to consider suspected shunts (liver, vascular connections, mesentery). For instance, if there is concern that the twins might be functionally the same from a circulatory standpoint, then it might make sense to give twice the medication dose to one twin.

Temperature regulation is a challenge in the delivery room resuscitation of all infants with congenital anomalies, but particularly in conjoined twins, where there is increased surface area and there may not be an ideal location for an overhead warmer [9]. Despite using an overhead warmer placed over the twins as best as possible with chemical mattresses under each twin, there was mild hypothermia in this cohort with a median first temperature of 36.2 °C but a reassuring final temperature of 36.4 °C in twin A and 36.5 °C in twin B. We have implemented quality improvement methodology to improve rates of euthermia overall and this remains an ongoing work in progress in our center [9].

There are also rare circumstances where one twin may be in extremis while the other twin is relatively unaffected and discussions should occur with the surgical team about the possibility for emergent separation. These discussions can be ethically complex and may benefit from employing an ethical framework to guide decision-making [10]. Indications to consider emergency separation include stillbirth in one twin [11], omphalocele rupture [12], gastroschisis [13], trauma to joint structures [14], and/or intestinal obstruction [15, 16]. If any of these scenarios are anticipated based on fetal surveillance, a second operating room should be prepared and staffed at delivery. In our large cohort, we had two instances of emergency separations, each with one surviving twin. In the first case, emergency separation was performed for severe hemodynamic instability with imminent demise in twin A. In the second case, twin B’s anomalies were incompatible with life, even if they remained conjoined, and so emergency separation was performed to save twin A. While emergency separations are rare, there are multiple instances in the literature. Ramlan et al. reported three emergency separations performed for worsening clinical status in one twin, 1) necrotizing enterocolitis with frequent desaturations and seizures at 12 days of life, 2) sepsis at 110 days of life, and 3) concern for a heteropagus twin, with ultimate demise in all six infants following emergency separation [17]. Burhamah et al. reported a case of omphalopagus twins born at 26 weeks’ gestation in which there was a ruptured omphalocele. Recovery was overall uncomplicated with ostomies followed by definitive surgical repairs in both twins by 1 year of age [18].

Among 19 pairs of conjoined twins, our team predicted prenatally that separation would be possible in nine pairs and potentially possible in an additional three pairs. Following active resuscitation of 13 sets of conjoined twins including nine sets deemed separable, two sets deemed potentially separable, and two sets deemed non-separable, there were 86.9% (*N* = 10) who had successful separation surgeries with 80% survival in twin A and 100% survival in twin B. Among the three sets not separated, two sets were deemed non-separable prenatally: 1) parapagus twins with only two lungs where twin B’s aorta gave rise to a large feeding vessel which was connected into the aorta of twin A who had a very small primitive heart and 2) thoracopagus twins with a shared, multichamber heart. The third set was considered potentially separable prenatally but ultimately deemed non-separable postnatally: 3) thoracopagus twins with cardiac failure in one twin and absence of venous liver drainage in the other twin with multiorgan failure in both.

For the six pairs of conjoined twins who had a planned non-intervention delivery, the team had determined prenatally that five sets were non-separable, and one set was potentially separable. At our center, families carrying a pregnancy with a suspected life-limiting diagnosis meet with our perinatal palliative care team for a complex antenatal resuscitation evaluation which consists of a maternal fetal medicine specialist, a neonatologist, a genetic counselor, an advanced practice provider, and the psychosocial team consisting of psychology, social work, and if desired, spiritual care, child life therapy, creative arts therapy, and psychiatry [19]. This multidisciplinary team meets with the family to develop an individualized plan to honor the birth and death of their child(ren). These infants typically remain in the room with their parents for at least the first 48 h of life to allow for maximal family bonding and memory making. If infants survive > 48 h, they are transferred to the NICU to coordinate additional care planning, including potential discharge planning with hospice if indicated.

For the 10 sets of twins who underwent separation, survival to NICU discharge was relatively high at 80% (*N* = 8/10) in twin A and 100% (*N* = 10/10) in twin B. In a large systematic review of 158 sets of thoracopagus conjoined twins, separation was predicted to be possible in 82 sets with an overall survival rate of about 50%. Among the remaining 71 sets deemed non-operable, all infants subsequently expired [20]. While separation and survival were higher in our cohort, we also saw demise in all infants not separated. In another large single-center review spanning 30 years, surgeons separated eight sets of conjoined twins, resulting in three deaths and an overall survival rate of 81%, comparable to our cohort [21].

Finally, the hospital journey of conjoined twins is often close to a year and requires multidisciplinary involvement from pediatric surgery, neonatology, maternal fetal medicine, plastic surgery, cardiology, pulmonology, cardiothoracic surgery, pediatric anesthesiology, otolaryngology, urology, nephrology, pediatric intensive care, radiology, nutrition, social work, psychology, developmental therapy, nursing, and respiratory therapy, among others, who are each essential in the successful management, separation, and discharge of conjoined twins.

Unique strengths of this report include that we describe management and outcomes for one of the largest cohorts of conjoined twins from over a decade at a large quaternary care institution. Limitations inherent to this population exist. In particular, this was an observational cohort study and therefore a non-randomized sample. As a single center study, outcomes may also reflect center-specific practice management differences. Given the overall rarity of conjoined twins, delivery room practices are not always represented in the literature; this study fills an important evidence gap specific to this high-risk population. As each delivery presents unique challenges, simulation is essential, not only to plan for the specific circumstances of each set of conjoined twins but also to prepare all team members, many of whom may have no prior experience with such deliveries. Teams should proactively anticipate unexpected challenges through detailed discussions of spatial constraints; performance and documentation of interventions; respiratory management, including the use of video laryngoscopes and laryngeal masks; establishment of intravenous access; and accurate weight estimation and medication dosing.

## Conclusion

The delivery room resuscitation of conjoined twins is complex with high rates of advanced respiratory intervention. These deliveries necessitate an experienced and cohesive multidisciplinary team as well as an individualized delivery plan based on specific anatomy and reinforced with simulation. Survival is high among twins who undergo separation.

## Data Availability

The data that support the findings of this study are not publicly available due to privacy reasons but are available from the corresponding author upon reasonable request.
